# Immunogenicity and safety of three aluminium hydroxide adjuvanted vaccines with reduced doses of inactivated polio vaccine (IPV-Al) compared with standard IPV in young infants in the Dominican Republic: a phase 2, non-inferiority, observer-blinded, randomised, and controlled dose investigation trial

**DOI:** 10.1016/S1473-3099(17)30177-9

**Published:** 2017-07

**Authors:** Luis Rivera, Rasmus S Pedersen, Lourdes Peña, Klaus J Olsen, Lars V Andreasen, Ingrid Kromann, Pernille I Nielsen, Charlotte Sørensen, Jes Dietrich, Ananda S Bandyopadhyay, Birgit Thierry-Carstensen

**Affiliations:** a Statens Serum Institut (SSI), Copenhagen, Denmark; b AJ Vaccines A/S, Copenhagen, Denmark; c Hospital Maternidad Nuestra Señora de la Altagracia, Santo Domingo, Dominican Republic; d Larix A/S, Herlev, Denmark; e Bill & Melinda Gates Foundation, Seattle, WA, USA

## Abstract

**Background:**

Cost and supply constraints are key challenges in the use of inactivated polio vaccine (IPV). Dose reduction through adsorption to aluminium hydroxide (Al) is a promising option, and establishing its effectiveness in the target population is a crucial milestone in developing IPV-Al. The aim of this clinical trial was to show the non-inferiority of three IPV-Al vaccines to standard IPV.

**Methods:**

In this phase 2, non-inferiority, observer-blinded, randomised, controlled, single-centre trial in the Dominican Republic, healthy infants aged 6 weeks, not previously polio vaccinated, were allocated after computer-generated randomisation by block-size of four, to receive one of four IPV formulations (three-times reduced dose [1/3 IPV-Al], five-times reduced dose [1/5 IPV-Al], ten-times reduced dose [1/10 IPV-Al], or IPV) intramuscularly in the thigh at 6, 10, and 14 weeks of age. The primary outcome was seroconversion for poliovirus types 1, 2, and 3 with titres more than or equal to four-fold higher than the estimated maternal antibody titre and more than or equal to 8 after three vaccinations. Non-inferiority was concluded if the lower two-sided 90% CI of the seroconversion rate difference between IPV-Al and IPV was greater than −10%. The safety analyses were based on the safety analysis set (randomly assigned participants who received at least one trial vaccination) and the immunogenicity analyses were based on the per-protocol population. This study is registered with ClinicalTrials.gov registration, number NCT02347423.

**Findings:**

Between Feb 2, 2015, and Sept 26, 2015, we recruited 824 infants. The per-protocol population included 820 infants; 205 were randomly assigned to receive 1/3 IPV-Al, 205 to receive 1/5 IPV-Al, 204 to receive 1/10 IPV-Al, and 206 to receive IPV. The proportion of individuals meeting the primary endpoint of seroconversion for poliovirus types 1, 2, and 3 was already high for the three IPV-Al vaccines after two vaccinations, but was higher after three vaccinations (ie, after completion of the expanded programme of immunisation schedule): 1/3 IPV-Al 98·5% (n=202, type 1), 97·6% (n=200; type 2), and 99·5% (n=204, type 3); 1/5 IPV-Al: 99·5% (n=204, type 1), 96·1% (n=197, type 2), and 98·5% (n=202, type 3); and 1/10 IPV-Al: 98·5% (n=201, type 1), 94·6% (n=193, type 2), and 99·5% (n=203, type 3). All three IPV-Al were non-inferior to IPV, with absolute differences in percentage seroconversion for each poliovirus type being greater than −10% (1/3 IPV-Al type 1, −1·46 [–3·60 to 0·10], type 2, −0·98 [–3·62 to 1·49], and type 3, −0·49 [–2·16 to 0·86]; 1/5 IPV-Al type 1, −0·49 [–2·16 to 0·86], type 2, −2·45 [–5·47 to 0·27], and type 3, −1·46 [–3·60 to 0·10]; and 1/10 IPV-Al type 1, −1·47 [–3·62 to 0·10], type 2, −3·94 [–7·28 to −0·97], and type 3, −0·49 [–2·17 to 0·86]). Three serious adverse events occurred that were unrelated to the vaccine.

**Interpretation:**

The lowest dose (1/10 IPV-Al) of the vaccine performed well both after two and three doses. Based on these results, this new vaccine is under investigation in phase 3 trials.

**Funding:**

Bill & Melinda Gates Foundation.

## Introduction

Eradication of poliomyelitis is in its final phase and several vaccination policy changes are being implemented to complete and sustain eradication according to the polio eradication and endgame strategic plan of the Global Polio Eradication Initiative.^1^ A major component of this plan is to expand the use of inactivated polio vaccine (IPV), especially in low-resource countries, as a replacement for oral polio vaccine (OPV) in the future.^2^, ^3^ Since April, 2016, these countries are in a transition phase in which bivalent OPV with only poliovirus types 1 and 3, and a supplementary dose of trivalent IPV, have replaced trivalent OPV for routine immunisation and supplemental immunisation activities. This shift in polio vaccination practices is supported by the results from several clinical trials investigating new bivalent OPV and IPV combination schedules.^4^, ^5^, ^6^, ^7^, ^8^ To succeed with this transition, the supply and cost constraints of IPV urgently need to be overcome. Many initiatives are ongoing to meet the increasing demand for IPV.^9^ One potential approach is the reduction of the amount of antigen per vaccine dose by up to five times, through intradermal administration with needle-syringe or needle-free devices. Some of the reported results are promising, both when the expanded programme of immunisation (EPI) schedule was employed,^10^, ^11^ and even more so when the doses were administered at older ages.^12^, ^13^, ^14^ Statens Serum Institut (SSI; Copenhagen, Denmark) has developed three new reduced-dose IPV-formulations adsorbed to aluminium hydroxide (IPV-Al) for intramuscular administration: three-times reduced dose (1/3 IPV-Al), five-times reduced dose (1/5 IPV-Al), and ten-times reduced dose (1/10 IPV-Al). On the basis of preclinical studies^15^ we anticipated that up to ten-times reduction of the antigen doses of each of the three poliovirus types in IPV was feasible, without substantially compromising the immunogenicity of the vaccine.

**Research in context**

**Evidence before this study**

We searched PubMed using the keywords “inactivated poliovirus vaccine”, “polio vaccination”, and “clinical trial”, for papers published between Jan 1, 2007, and Jan 31, 2017. The safety and immunogenicity of inactivated polio vaccine (IPV) adsorbed to aluminium hydroxide (IPV-Al) is well established through a long track record of worldwide clinical use in childhood vaccination programmes. Thus, no safety concerns were anticipated for the three new vaccines. In a recent trial in adolescents, the safety of the three IPV-Al vaccines was supported and they all induced robust anamnestic responses as booster vaccines. Our trial was the first to investigate the non-inferiority of the immunogenicities of the three IPV-Al vaccines, compared with a standard dose of IPV, when administered as primary vaccinations to young infants. In previous clinical trials under similar conditions, five-times dose-reduced non-adjuvanted IPV was administered intradermally either by a needle-free device or by the Mantoux injection technique, and compared with standard IPV administered intramuscularly.

**Added value of this study**

The results from this trial show the non-inferiority of the immunogenicity of three new IPV-Al vaccines compared with IPV in the context of primary vaccination in infants. These results show the feasibility, in infants, of substantially (up to ten-times) reducing the antigen doses of the three poliovirus types in a stand-alone IPV formulation containing aluminium hydroxide. The non-adjuvanted comparator, IPV, contained standard doses of 40 (type 1), 8 (type 2), and 32 (type 3) D-antigen units. The seroconversion rates for the three new IPV-Al vaccines and IPV were high. On the basis of the results reported here, IPV-Al is now under investigation in phase 3 trials.

**Implications of all the available evidence**

The results of this trial should be a promising first step towards optimising the use of IPV in the context of growing supply constraints. If the results of the planned phase 3 investigations are favourable, and followed by regulatory approval and WHO prequalification, the available supply of IPV in the world could be increased, contributing substantially to the global polio eradication programme. IPV-Al is a stand-alone vaccine that could be used as a replacement for oral polio vaccine, both for routine immunisation and for supplemental immunisation activities.

In a first-in-human, proof-of-concept trial, the three new IPV-Al vaccines were investigated when given as a booster dose. The enrolled Danish adolescents, who were 10–15 years old, had a history of vaccination with IPV at 3, 5, and 12 months, and 5 years. The three IPV-Al vaccines were safe and all induced robust anamnestic responses as booster doses.^16^ In the clinical trial reported here, the objective was to show the non-inferiority of the three vaccines compared with standard IPV, in infants vaccinated according to a primary vaccination schedule of 6, 10, and 14 weeks of age, as per the EPI. This is a challenging schedule for IPV, given the early age of the first vaccination and the short intervals between the doses, because high levels of maternal antibodies interfere with IPV immunogenicity.^17^ The aim of investigating the new vaccines with this primary vaccination schedule was to achieve clinical evidence of the immunogenicity and safety of the new IPV-Al vaccines that would be applicable for low-resource countries, including countries using the EPI schedule, because these countries are potential target countries for IPV-Al.

## Methods

### Study design and participants

The study was a phase 2, non-inferiority, observer-blinded, randomised, parallel, and controlled dose investigation trial, with three investigational IPV-Al groups and one IPV comparator group. For all groups, the trial consisted of four visits. Visit 1, when an infant was aged 6 weeks, included collection of informed consent and the first blood sample, screening, and first vaccination. At visit 2, when the infant was aged 10 weeks, the second vaccination was given. At visit 3, when the infant was aged 14 weeks, the second blood sample was collected, and the third vaccination given. At visit 4, when the infant was aged 18 weeks, a third blood sample was collected.

Trial participants were healthy 6-week-old infants in the Dominican Republic who had not previously received any polio vaccination (OPV or IPV). Participants were recruited between Feb 2, 2015, and Sept 26, 2015. The trial participants were recruited at the Centre for Neonatal Research, Hospital Maternidad Nuestra Senora de la Altagracia, Santo Domingo, Dominican Republic. The most important exclusion criteria included exposure to OPV in the household, defined as known exposure to OPV or poliovirus in household in 3 months before inclusion or planned OPV in the household during the trial; and known or suspected immune deficiency or family history of congenital or hereditary immune deficiency, severe uncontrolled chronic disease, and known or suspected allergy to vaccine constituents. All participants were recruited by contacting parents who were visiting the maternity ward. Participants were randomly allocated into the four groups of 1/3 IPV-Al, 1/5 IPV-Al, 1/10 IPV-Al, and IPV.

Before inclusion of the first trial participant, the ethics committee of Ministerio de Salud Pública y Asistencia Social Hospital de Maternidad Nuestra Señora de la Altagracia Comite de Bioética, Santo Domingo, and the authority of Ministerio de Salud Pública, CONABIOS Consejo Nacional de Bioética en Salud, Avenida Bolivar (No. 902), La Julia, Santo Domino (No. CONABIOS 049-2014) approved the clinical trial application (EudraCT No: 2014-003449-88). The trial was conducted according to good clinical practice and the current version of the Declaration of Helsinki, adopted on the 64th World Medical Association General Assembly, October, 2013.

### Randomisation and masking

The two vaccine formulations (IPV-Al *vs* IPV) were visually distinguishable. The observer blinding of the trial ensured that only prespecified unblinded study staff and the trial monitor had access to the trial vaccines and the dispensing logs, and that only the unblinded study staff were present during the administration of the trial vaccines. The remaining trial staff, including the investigators assessing the adverse events, the clinical trial manager, and the laboratory staff at SSI determining the antibody titres, were blinded until database lock and release. Randomisation lists (block-randomisation, block-size of four) were generated by a validated SAS program (version 9.3) by a statistician, who was not in any way involved in the statistical or data management plans for the trial. The lists were kept in a restricted access folder, and individually sealed randomisation envelopes were prepared and distributed to the investigational site. At the site, a new participant was allocated the lowest available participant (randomisation) number. By opening the randomisation envelope with the corresponding number, the unblinded staff responsible for allocation or administration of the trial vaccine could identify the vaccine to be given to the participant. The principal investigator could at any time unblind through an emergency unblinding procedure, if this was needed to ensure the continued safety of an infant.

### Trial vaccines and administration

The trial vaccines were all stand-alone trivalent IPV containing inactivated poliovirus type 1 (Brunhilde), type 2 (MEF-1), and type 3 (Saukett). The declared amounts of the poliovirus types in the comparator vaccine (IPV) was 40 D-antigen units (DU) for type 1, 8 DU for type 2, and 32 DU for type 3, per dose of 0·5 mL. These are standard doses in use by all IPV manufacturers. For poliovirus type 1, other IPV manufacturers use the Mahoney strain (40 DU). In the three investigational reduced-dose vaccines, the corresponding declared amounts of poliovirus types 1, 2, and 3 per dose were 13·3 DU (type 1), 2·7 DU (type 2), and 10·7 DU (type 3) for 1/3 IPV-Al; 8 DU (type 1), 1·6 DU (type 2), and 6·4 DU (type 3) for 1/5 IPV-Al); and 4 DU (type 1), 0·8 DU (type 2), and 3·2 DU (type 3) for 1/10 IPV-Al. All formulations maintained the ratios between the three poliovirus types, and contained aluminium hydroxide, corresponding to 0·5 mg aluminium per dose of 0·5 mL. The comparator (IPV) was a clear solution for injection, whereas the three investigational vaccines (IPV-Al) were suspensions for injection. All vaccines were stored between 2°C and 8°C at the investigational sites, with monitoring of storage conditions. The four vaccines were administered as 0·5 mL intramuscular injections in the anterolateral aspect of the left thigh by use of a syringe fitted with a 23 gauge, 25 mm needle. Concomitant vaccines were administered outside of the trial in the opposite thigh, according to the Dominican Republic vaccination schedule (except for polio vaccination). At the end of the trial, all trial participants were referred to the national vaccination system to receive two extra doses of OPV at approximately 18 and 26 weeks of age, to offer adequate protection against polio to all infants, irrespective of the performance of the investigated trial vaccines.

### Outcomes

The primary immunogenicity outcome was the rate of seroconversion for poliovirus types 1, 2, and 3 with titres more than or equal to four-fold higher than the estimated maternal antibody titre and more than or equal to a titre of 8 measured 4 weeks after the third vaccination for each vaccine. The secondary outcomes were type-specific geometric mean titres 4 weeks after the third vaccination for each vaccine, type-specific seroprotection rates (titres more than or equal to an eighth) 4 weeks after the third vaccination for each vaccine, type-specific reverse cumulative titre distribution curves based on prevaccination and 4 weeks post-third vaccination serum titres for each vaccine, and all adverse events following the vaccinations for each vaccine. Non-inferiority of the IPV-Al vaccines was concluded if the lower two-sided 90% CI of the seroconversion rate difference between IPV-Al and IPV was greater than −10%.

### Safety assessments

The infants were observed for 30 min after each vaccination and immediate adverse events were recorded. A diary, thermometer, and ruler were given to parents for daily recording and measuring of injection site reactions, temperature reactions, and other solicited adverse events during the first 3 days (72 h) after vaccination, and for recording of any adverse event during the 7 days after vaccination. The solicited events in the diary were injection site redness or swelling reactions, axillary temperatures, persistent crying for more than 3 h, irritability, drowsiness, loss of appetite, vomiting, and diarrhoea. Parents also recorded use of concomitant medications. All adverse events were assessed for seriousness, relatedness to the vaccine, intensity, and outcome, and were transferred to the electronic case record form. Adverse events were coded by MedDRA, MSSO version 17.1. A data safety monitoring board comprised independent experts who advised the investigators and sponsors during the active phase of the trial and made recommendations regarding continuation, modification, or termination of the trial. The data safety monitoring board held two evaluation meetings. The recommendation from the board after both meetings was to continue the trial without modifications.

### Immunogenicity assessments

The Vero cell assay was used for determination of neutralising antibodies against poliovirus types 1, 2, and 3, essentially as described by Melnick and colleagues.^18^ The assay was done at SSI in Denmark and was fully validated. Each well of two 96-well microtitre plates was filled with 50 μL of incubation medium. 50 μL of a serum sample was added to the first well in two rows on the first plate, and a two-fold dilution series was generated over two plates. Following this, approximately 100 cell culture infectious dose 50% (CCID_50_) of poliovirus types 1, 2, or 3 in 50 μL incubation medium was added to each well, keeping just one poliovirus type per plate. The microtitre plates were incubated on a shaker in an incubator at 37°C (range 36–38°C), 5% (4–6%) CO_2_ for 5·5 (4–6) h. Afterwards, the plates were incubated at 2–8°C (average 5°C) for 18–22 h. After incubation, 50 μL of Vero cell suspension (60 000 cells/mL) was added to all wells in each plate. The plates were sealed with plate-sealing tape and incubated at 37°C (range 36–38°C), 5% (4–6%) CO_2_ for 7 (6–8) days. The wells showing neutralising or cytopathogenic activity were recorded. The result from a single dilution series was given as ([1/√2] × the lowest dilution factor with dead Vero cells, and the final titre calculated as the geometric mean of the results from the two independent dilution series. The lower limit in the measurable range of titres was 1·4 and there was no upper limit.

### Statistical analysis

The sample size calculations were based on evaluating different scenarios for the seroconversion rates for the three poliovirus types, considering seroconversion rates for the different poliovirus types of: 89% (type 1), 96% (type 2), and 99% (type 3);^11^ and 86% (type 1), 86% (type 2), and 97% (type 3)^19^ from published clinical trials, in which standalone IPV was investigated under similar conditions. The power heavily depended on the seroconversion rates, particularly if they were less than 90%. With a 5% one-sided type 1 error level, and assuming the same true seroconversion rates in full-dose and reduced-dose treatments, 200 participants per group gave powers of 94%^11^ and 80%^19^ for the two scenarios mentioned above. On the basis of these considerations, we decided to include 200 evaluable participants per group with six additional participants per group to allow for possible 3% dropout, estimated on the basis of previous experience for similar trials at the same investigational site. The safety analysis set was defined as randomised participants who received at least one trial vaccination. The full analysis set was defined as participants who received at least one trial vaccination and had at least one post-baseline immunogenicity measurement, where the per-protocol population was defined as the full analysis set with no major protocol deviations. The statistical analysis plan was signed before the first participant's first visit. The safety analyses were based on the safety analysis set and the immunogenicity analyses were based on the per-protocol population. The primary analyses were repeated for the full analysis set. For each individual and poliovirus type, the primary seroconversion endpoint was positive if, at 4 weeks after the third vaccination, the type-specific titre was greater than or equal to 8, and greater than or equal to four-times the estimated maternal antibody titre.

Otherwise, the results would be negative. If at least one of the two criteria could not be evaluated, the seroconversion endpoint would remain missing. The estimated maternal antibody titre was calculated as:

$$\text{Titre}\;(\text{t})=\text{Titre}\;(\text{base})\times \exp\;\left(-\frac{\ln(2)}{{\text{t}}_{1/2}}\times \text{t}\right)$$

where t is the time since baseline (days) and t_1/2_ is the expected half-life of maternal antibodies of 28 days. The null hypothesis for each endpoint was (P_i,j,red_<P_i,full_)–10%, where P_i,j,red_ was the seroconversion rate for poliovirus type i (i=1, 2, or 3) and reduced dose j (j=1/3 IPV-Al, 1/5 IPV-Al, or 1/10 IPV-Al), and P_i,full_ was the seroconversion rate for IPV and poliovirus type 1. The 10% signified 10 percentage points. The alternative hypothesis (H_1ij_) was (P_i,j, red_≥P_i,full_)–10%.

If H_0ij_ was rejected, this would have indicated non-inferiority for one IPV-Al vaccine and poliovirus type. However, we drew conclusions only on the treatment level: for a reduced dose (j) to be concluded non-inferior, all three hypotheses (H_01j_, H_02j_, and H_03j_) had to be rejected. Conclusions were made independently for each IPV-Al vaccine. Each of the primary analyses was evaluated by calculating the unadjusted rate difference (P_i,j,red_–P_i,full_) with a two-sided 90% CI. The CIs were calculated as approximative Newcombe-Wilson intervals. The H_0_ was to be rejected if the CI was fully above the −10% limit and this, therefore, corresponded to a one-sided test at a 5% confidence level. Multiplicity adjustments were not introduced, since non-inferiority for each IPV-Al vaccine was concluded only when it had been shown for all three poliovirus types, and since each of the three IPV-Al treatments were assessed independently.

In the geometric mean titre calculations for the secondary outcomes, the geometric mean value of two dilution series was used. Data were received as log titre values. For datapoints measured as the lower limit of the measurable range, these values were used directly in the statistical calculations. The mean for each IPV-Al vaccine and the difference from IPV was back-transformed to yield geometric mean titre values and ratios with 95% CI. In an exploratory immunogenicity analysis 4 weeks after the second vaccination, the primary and secondary immunogenicity endpoints defined previously were repeated without formal comparisons to the non-inferiority limits. SAS version 9.3 was used for the data processing and reporting in this trial.

This study is registered with ClinicalTrials.gov registration, number NCT02347423.

### Role of the funding source

The Bill & Melinda Gates Foundation contributed scientific support to the trial design. The funder of the study had no role in data collection, data analysis, data interpretation, or writing of the report. The findings and conclusions in this publication are those of the authors. All authors had access to the data from the trial, and share the responsibility for the decision to submit for publication.

## Results

One of the 824 randomly assigned and vaccinated individuals in the safety analysis set discontinued the trial after the first vaccination because of withdrawal of consent to participate. Of the 823 participants in the full analysis set, three individuals across the groups had major protocol deviations and were excluded from the per-protocol population (figure). The mean age at inclusion was approximately 44 days (SD 2·1), with no major differences in age, sex, height, and birthweight across the four groups (table 1). As expected, a high proportion of the participants were seroprotected (titre ≥8) at baseline by maternal antibodies against one or more of the three poliovirus types: 498 (61%) participants for type 1, 737 (90%) participants for type 2, and 466 (57%) participants for type 3 in the total per-protocol population.

**Figure:**
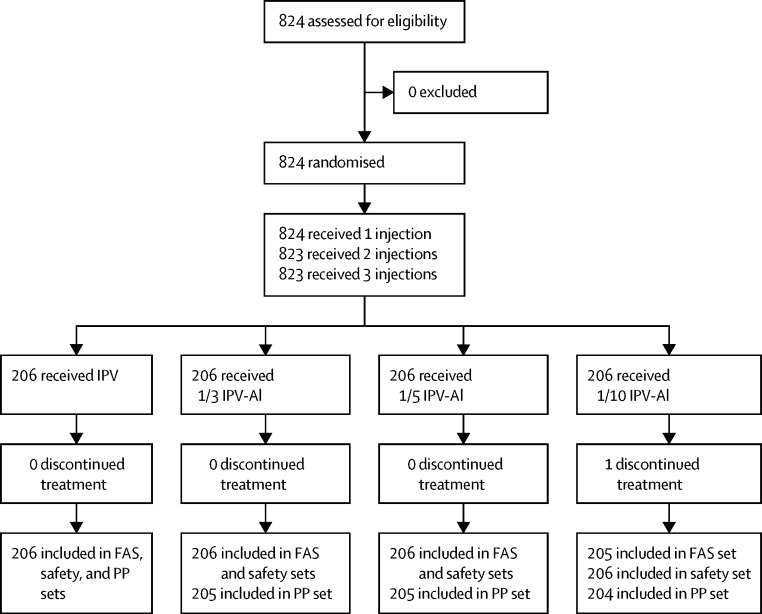
Trial profile All vaccines were manufactured by SSI. FAS=full analysis set. IPV=inactivated polio vaccine. IPV-Al=IPV adsorbed to aluminium hydroxide. PP=per-protocol.

**Table 1:** Baseline characteristics of the full analysis set

		**IPV (n=206)**	**1/3 IPV-Al (n=206)**	**1/5 IPV-Al (n=206)**	**1/10 IPV-Al (n=206)**
Sex					
	Female	109 (52·9)	99 (48·1)	100 (48·5)	109 (52·9)
	Male	97 (47·1)	107 (51·9)	106 (51·5)	97 (47·1)
Age (days)		44·3 (2·1)	44·0 (2·1)	44·2 (2·1)	44·0 (2·1)
Height (cm)		55·4 (3·0)	55·1 (2·3)	55·2 (2·5)	55·0 (2·6)
Weight (g)		4797 (581)	4743 (502)	4831 (541)	4732 (557)
Birthweight (g)		3195 (407)	3225 (381)	3236 (412)	3180 (386)

Data are n (%) or mean (SD), unless otherwise indicated. Data are for number of participants in the safety analysis set. IPV=inactivated polio vaccine. IPV-Al=IPV adsorbed to aluminium hydroxide.

The three new IPV-Al vaccines were non-inferior to IPV, for poliovirus types 1, 2, and 3 in the primary immunogenicity analysis, indicated by the lower two-sided 90% CIs of seroconversion rate differences being above the predefined delta value of −10% (table 2). In an exploratory analysis, non-inferiority could also be shown for the three new vaccines compared with IPV when using two-sided 95% CIs (table 2). For all poliovirus types, the postvaccination geometric mean titres increased with increasing antigen dose. Furthermore, the geometric mean titres increased considerably from 4 weeks after the second vaccination (visit 3) to 4 weeks after the third vaccination (visit 4; Table 3, Table 4, Table 5). The proportion of individuals meeting the primary endpoint of seroconversion was high for the three IPV-Al vaccines after three vaccinations—ie, after completion of the EPI schedule (Table 3, Table 4, Table 5). The lowest seroconversion rate was 75·0% for poliovirus type 2 after two vaccinations with 1/10 IPV-Al, which increased to 94·6% after three vaccinations. All IPV-Al vaccines induced seroconversion for poliovirus types 1 and 3 in more than 89% of the infants after two vaccinations, and in more than 98% after three vaccinations. Four infants had a titre less than 8 one month after their last vaccination: two participants for the type 1 poliovirus type, who received 1/3 IPV-Al and 1/5 IPV-Al, and two participants for the type 3 poliovirus type who received 1/5 IPV-Al and 1/10 IPV-Al. The reverse cumulative titre distributions (appendix pp 1–3) illustrate that all vaccines were immunogenic with similar shapes of the distribution curves across poliovirus types and vaccines. Moreover, it is evident from the curves that for all poliovirus types, the distance between the IPV-Al and IPV curves increases with decreasing antigen dose of IPV-Al. The same pattern is more pronounced for the curves after only two vaccinations (data not shown). Overall the immunogenicity results from the trial show that all three reduced dose IPV-Al vaccines were highly immunogenic and non-inferior to IPV when administered to young infants using the EPI schedule.

**Table 2:** Results of the primary non-inferiority immunogenicity analysis

	**n (%; 90% CI)**	**Difference *vs* IPV (90% CI)**	Difference *vs* IPV (95% CI)*
**IPV (n=206)**			
Type 1	206 (100%; 98·6 to 100)	NA	NA
Type 2	203 (98·5%, 96·3 to 99·6)	NA	NA
Type 3	206 (100%, 98·6 to 100)	NA	NA
**1/3 IPV-Al (n=205)**			
Type 1	202 (98·5%, 96·3 to 99·6)	−1·46 (−3·60 to 0·10)	−1·46 (−4·21 to 0·61)
Type 2	200 (97·6%, 94·9 to 99·0)	−0·98 (−3·62 to 1·49)	−0·98 (−4·27 to 2·09)
Type 3	204 (99·5%, 97·7 to 100)	−0·49 (−2·16 to 0·86)	−0·49 (−2·71 to 1·39)
**1/5 IPV-Al (n=205)**			
Type 1	204 (99·5%; 97·7 to 100)	−0·49 (−2·16 to 0·86)	−0·49 (−2·71 to 1·39)
Type 2	197 (96·1%; 93·1 to 98·0)	−2·45 (−5·47 to 0·27)	−2·45 (−6·18 to 0·89)
Type 3	202 (98·5%; 96·3 to 99·6)	−1·46 (−3·60 to 0·10)	−1·46 (−4·21 to 0·61)
**1/10 IPV-Al (n=205)**			
Type 1	201 (98·5%; 96·2 to 99·6)	−1·47 (−3·62 to 0·10)	−1·47 (−4·23 to 0·60)
Type 2	193 (94·6%; 91·2 to 96·9)	−3·94 (−7·28 to −0·97)	−3·94 (−8·05 to −0·33)
Type 3	203 (99·5%; 97·7 to 100)	−0·49 (−2·17 to 0·86)	−0·49 (−2·72 to 1·38)

Individual CIs with Clopper-Pearson intervals. Difference presented with Newcombe score CIs. IPV=inactivated polio vaccine. IPV-Al=IPV adsorbed to aluminium hydroxide. NA=not applicable.

* Data from exploratory post-second vaccination analysis; 95% CIs.

**Table 3:** Immunogenicity results for poliovirus type 1

	**1/3 IPV-Al (n=205)**	**1/5 IPV-Al (n=205)**	**1/10 IPV-Al (n=204)**	**IPV (n=206)**	**Total (n=820)**
**Prevaccination visit 1**					
Mean (GMT)	13·0 (10·2–16·4)	10·4 (8·5–12·9)	12·7 (10·1–15·8)	14·4 (11·5–18·1)	12·5 (11·2–14·0)
Median	11·3	11·3	11·3	16·0	11·3
Seroprotection*	125 (61·0%)	120 (58·5%)	125 (61·3%)	128 (62·1%)	498 (60·7%)
**Post-second vaccination visit 3 (exploratory)**					
Mean (GMT)	1030·9 (778·7–1364·9)	637·8 (476·0–854·7)	432·0 (330·9–564·0)	2557·3 (2091·0–3127·5)	NA
Median	1448·2	724·1	512·0	2896·3	NA
Seroprotection*	200 (97·6%)	196 (95·6%)	195 (95·6%)	206 (100·0%)	NA
Seroconversion (primary outcome)†	187 (91·2%)	188 (91·7%)	182 (89·2%)	197 (95·6%)	NA
Seroconversion‡	190 (92·7%)	197 (96·1%)	191 (93·6%)	197 (95·6%)	NA
**Post-third vaccination visit 4**					
Mean (GMT)	3310·2 (2738·2–4001·5)	2221·1 (1808·9–2727·3)	1584·6 (1277·8–1965·1)	3727·7 (3211·1–4327·4)	NA
Median	4096·0	2896·3	1448·2	4096·0	NA
Seroprotection*	204 (99·5%)	204 (99·5%)	204 (100%)	206 (100%)	NA
Seroconversion (primary outcome)†	202 (98·5%)	204 (99·5%)	201 (98·5%)	206 (100%)	NA
Seroconversion‡	203 (99·0%)	204 (99·5%)	201 (98·5%)	206 (100%)	NA

Data are for per-protocol population. IPV=inactivated polio vaccine. IPV-Al= IPV adsorbed to aluminium hydroxide. GMT=geometric mean concentration. NA=not applicable.

* Titre ≥8.

† Titre ≥4 × estimated maternal antibody titre and titre ≥8.

‡ Titre ≥4 × estimated maternal antibody titre.

**Table 4:** Immunogenicity results for poliovirus type 2

	**1/3 IPV-Al (n=205)**	**1/5 IPV-Al (n=205)**	**1/10 IPV-Al (n=204)**	**IPV (n=206)**	**Total (n=820)**
**Prevaccination visit 1**					
Mean (GMT)	47·4 (38·3–58·7)	46·4 (38·4–56·1)	51·6 (41·0–64·9)	53·7 (43·8–65·9)	49·7 (44·8–55·2)
Median	45·3	45·3	45·3	45·3	45·3
Seroprotection*	183 (89·3%)	190 (92·7%)	180 (88·2%)	184 (89·3%)	737 (89·9%)
**Post-second vaccination visit 3 (exploratory)**					
Mean (GMT)	1495·4 (1146·6–1950·4)	1317·3 (1031·4–1682·6)	646·2 (496·4–841·1)	2209·1 (1752·1–2785·3)	NA
Median	2048·0	1448·2	724·1	2896·3	NA
Seroprotection*	205 (100%)	205 (100%)	204 (100%)	206 (100%)	NA
Seroconversion (primary outcome)†	168 (82·0%)	173 (84·4%)	153 (75·0%)	181 (87·9%)	NA
Seroconversion‡	168 (82·0%)	173 (84·4%)	153 (75·0%)	181 (87·9%)	NA
**Post-third vaccination visit 4**					
Mean (GMT)	4495·1 (3726·5–5422·3)	3151·8 (2575·2–3857·4)	2410·8 (1956·3–2970·9)	3759·2 (3224·3–4382·8)	NA
Median	4096·0	4096·0	2896·3	4096·0	NA
Seroprotection*	205 (100%)	205 (100%)	204 (100%)	206 (100%)	NA
Seroconversion (primary outcome)†	200 (97·6%)	197 (96·1%)	193 (94·6%)	203 (98·5%)	NA
Seroconversion‡	200 (97·6%)	197 (96·1%)	193 (94·6%)	203 (98·5%)	NA

Data are for per-protocol population. IPV=inactivated polio vaccine. IPV-Al=IPV adsorbed to aluminium hydroxide. GMT=geometric mean concentration. NA=not applicable.

* Titre ≥8.

† Titre ≥4 × estimated maternal antibody titre and titre ≥8.

‡ Titre ≥4 × estimated maternal antibody titre.

**Table 5:** Immunogenicity results for poliovirus type 3

	**1/3 IPV-Al (n=205)**	**1/5 IPV-Al (n=205)**	**1/10 IPV-Al (n=204)**	**IPV (n=206)**	**Total (n=820)**
**Prevaccination visit 1**					
Mean (GMT)	10·3 (8·4–12·7)	10·3 (8·3–12·8)	11·2 (9·2–13·6)	11·9 (9·7–14·7)	10·9 (9·9–12·1)
Median	8·0	8·0	11·3	8·0	8·0
Seroprotection*	112 (54·6%)	115 (56·1%)	120 (58·8%)	119 (57·8%)	466 (56·8%)
**Post-second vaccination visit 3 (exploratory)**					
Mean (GMT)	1233·3 (917·2–1658·4)	971·7 (724·4–1303·4)	612·0 (442·9–845·7)	3702·7 (2916·4–4701·0)	NA
Median	2048·0	1448·2	1024·0	5792·6	NA
Seroprotection*	198 (96·6%)	199 (97·1%)	194 (95·1%)	203 (98·5%)	NA
Seroconversion (primary outcome)†	190 (92·7%)	191 (93·2%)	183 (89·7%)	201 (97·6%)	NA
Seroconversion‡	197 (96·1%)	195 (95·1%)	190 (93·1%)	202 (98·1%)	NA
**Post-third vaccination visit 4**					
Mean (GMT)	4229·7 (3537·6–5057·2)	3120·0 (2555·5–3809·2)	2069·0 (1638·8–2612·2)	4531·1 (3942·2–5207·9)	NA
Median	4096·0	4096·0	2896·3	4096·0	NA
Seroprotection*	205 (100%)	204 (99·5%)	203 (99·5%)	206 (100%)	NA
Seroconversion (primary outcome)†	204 (99·5%)	202 (98·5%)	203 (99·5%)	206 (100%)	NA
Seroconversion‡	204 (99·5%)	203 (99·0%)	203 (99·5%)	206 (100%)	NA

Data are for per-protocol population. IPV=inactivated polio vaccine. IPV-Al= IPV adsorbed to aluminium hydroxide. GMT=geometric mean concentration. NA=not applicable.

* Titre ≥8.

† Titre ≥4 × estimated maternal antibody titre and titre ≥8.

‡ Titre ≥4 × estimated maternal antibody titre.

Three serious adverse events, all assessed as being unrelated to the vaccinations, occurred in the trial: two events of bronchiolitis and one event of amoebic dysentery. 19 adverse events were reported in the trial including the abovementioned three serious adverse events. Three mild injection site reactions (≤25 mm) and 16 systemic adverse events, all with group frequencies less than 2%. This relatively low number of recorded adverse events was detected as a trend after inclusion of the first 100 infants in the trial, and led to reinforcement of the procedures for diary instructions to the parents and for adverse event recording in the electronic case record forms by the investigational site staff during the remaining active trial period. The trend was also noted by the data safety monitoring board.

## Discussion

The seroconversion (primary endpoint) rates for the different poliovirus types were 99% (type 1), 95% (type 2), and 100% (type 3) after completing the expanded programme of immunisation schedule for 1/10 IPV-Al, where the corresponding rates were 100% (type 1), 99% (type 2), and 100% (type 3) for IPV (Table 2, Table 3, Table 4, Table 5). In summary, the results from our trial strongly support a dose sparing strategy of three doses of aluminium hydroxide adjuvanted 1/10 IPV-Al using the EPI schedule. The immunogenicity of the lowest investigated dose, 1/10 IPV-Al, is non-inferior to standard IPV, despite the fact that it contains a tenth of a standard human dose of IPV. The seroconversion rates after two doses 1/10 IPV-Al were also considerably higher than those previously reported for two doses non-adjuvanted 1/5 IPV administered intradermally at 6 and 10 weeks.^9^, ^11^ Non-adjuvanted 1/5 IPV appears to perform very well when given as two intradermal doses at older ages (eg, 4 months and 8 months) or with larger dose intervals (eg, 4 months),^9^, ^12^, ^14^ or given as one intramuscular diphtheria, tetanus toxoids, whole-cell pertussis, hepatitis B, IPV, *Haemophilus influenzae* type b (DTwP-HepB-IPV/Hib) vaccination to toddlers.^20^ These results have paved the way for further clinical investigations of IPV-Al in phase 3 trials.

Previous clinical trials and post-marketing experience with the registered vaccines of IPV; diphtheria, tetanus, and pertussis (DTaP)-IPV; and tetanus, diphtheria, pertussis (TdaP)-IPV,^21^, ^22^, ^23^, ^24^, ^25^ all manufactured by SSI, and many years of worldwide clinical experience with aluminium adjuvanted IPV (manufactured by others), in various combination vaccines, support the finding that IPV and aluminium hydroxide are safe in combination.^17^, ^21^, ^26^ The first investigation of IPV-Al in adolescents supported this finding.^16^ The low frequency of adverse events in this phase 2 trial suggests that a safety evaluation is not necessarily justified. Thus, a complete safety evaluation of IPV-Al will have to await the safety results from ongoing phase 3 trials.

In other trials, reduced doses of 1/5 IPV (administered intradermally) as well as standard doses of IPV (administered intramuscularly) have been investigated in similar populations, also using the EPI schedule,^10^, ^11^, ^19^ in which both 1/5 IPV and IPV were non-adjuvanted. In these studies, seroconversion rates for poliovirus types 1, 2, and 3 after the vaccination schedule was completed ranged from 53% to 99% for 1/5 IPV (intradermally), and from 89% to 100% for IPV (intramuscularly)^10^, ^11^, ^19^ Although the seroconversion rates were high for 1/10 IPV-Al in this trial, the post-third vaccination geometric mean titres after vaccination with 1/10 IPV-Al were significantly lower than for IPV for poliovirus types 1, 2, and 3, respectively. However, with a protection threshold of a titre of 8, even the geometric mean titres after vaccination with the lowest dose in this trial might be adequate. The confirmatory phase 3 trials will include a long-term follow-up titre measurement 6 months after completion of the priming schedule to obtain information on long-term protection rates. A positive correlation between the antigen amount in the investigated vaccines and the post-third vaccination geometric mean titres (Table 3, Table 4, Table 5) was seen for all poliovirus types. In an exploratory analysis, seroconversion rates 1 month after the first two vaccinations (at 6 and 10 weeks) were reported (Table 3, Table 4, Table 5). Even for the two-dose assessment, 1/10 IPV-Al did well, with seroconversion rates of 89% (type 1), 75% (type 2), and 90% (type 3). These seroconversion rates are indeed higher than those reported from a Cuban trial^9^, ^11^ investigating two vaccinations of non-adjuvanted 1/5 IPV (at 6 and 10 weeks) administered intradermally by a needle-free device—21% (type 1), 55% (type 2), and 43% (type 3)—but comparable to two standard doses of IPV, administered intramuscularly (63% [type 1], 76% [type 2], and 93% [type 3]). In a trial in the Philippines,^10^ the seroconversion rates were 99% (type 1), 95% (type 2), and 95% (type 3) 1 month after completion of the EPI schedule, when administering non-adjuvanted 1/5 IPV intradermally by the Mantoux technique (comparable to the three IPV-Al vaccines; Table 3, Table 4, Table 5). An interpretation of these trials is that reduced doses of IPV can be injected either intradermally as non-adjuvanted formulations, or intramuscularly as aluminium hydroxide adjuvanted formulations, with similar immunogenicity results when completing the three-dose EPI schedule.

After two vaccinations at 6 and 10 weeks, the aluminium hydroxide-adjuvanted vaccines administered intramuscularly in our trial, were all more immunogenic than the non-adjuvanted 1/5 IPV administered intradermally in the Cuban trial.^11^ Seroconversion rates after injection of two non-adjuvanted 1/5 IPV doses intradermally by the Mantoux technique were not reported from the trial in the Philippines.^10^ The Vero cell assay was used in all these trials, but there are inter-assay differences, which is a limitation when comparing the results between the trials. In our trial Vero cells and wild-type viruses were used in the assay, versus HEp-2 cells and Sabin strains in other trials. This difference is known to affect the measured titre values.

The results of this phase 2 trial should be a first step to help achieve and sustain polio eradication in the long term by addressing the issue of availability of effective vaccine choices.
