# Supplementary material for: Immunogenicity and safety of three aluminium hydroxide adjuvanted vaccines with reduced doses of inactivated polio vaccine (IPV-Al) compared with standard IPV in young infants in the Dominican Republic: a phase 2, non-inferiority, observer-blinded, randomised, and controlled dose investigation trial
Source: Lancet Infect Dis. 2017 Jul;17(7):745–53. doi: 10.1016/S1473-3099(17)30177-9 (PMC5483484; doi:10.1016/S1473-3099(17)30177-9)
Supplement: Supplementary appendix [file mmc1.pdf]

THE LANCET Infectious Diseases

Supplementary webappendix

This webappendix formed part of the original submission and has been peer reviewed. We post it as supplied by the authors.

Supplement to: Rivera L, Pedersen RS, Peña L, et al. Immunogenicity and safety of three aluminium hydroxide adjuvanted vaccines with reduced doses of inactivated polio vaccine (IPV-Al) compared with standard IPV in young infants in the Dominican Republic: a phase 2, non-inferiority, observer-blinded, randomised, and controlled dose investigation trial. *Lancet Infect Dis* 2017; published online April 25. http://dx.doi.org/10.1016/S1473-3099(17)30177-9.

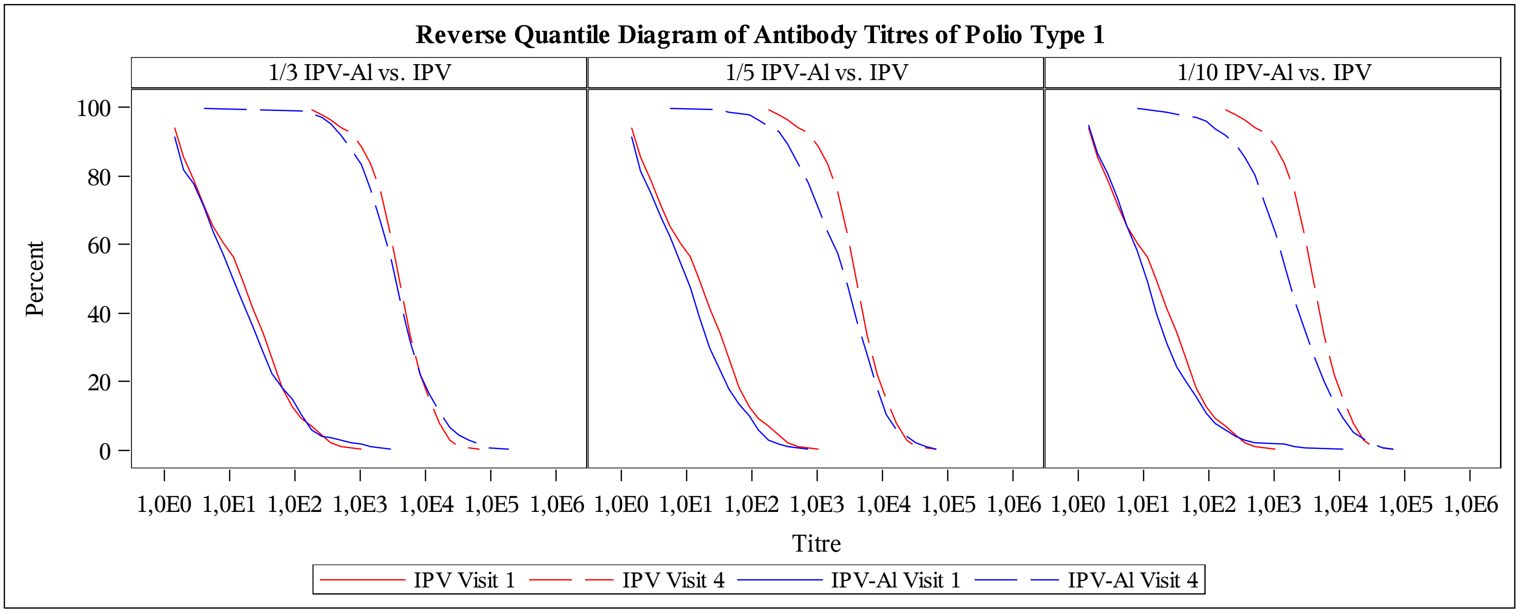
Reverse Quantile Diagram of Antibody Titres of Polio Type 1
1/3 IPV-Al vs. IPV
1/5 IPV-Al vs. IPV
1/10 IPV-Al vs. IPV
Percent
100
80
60
40
20
0
1,0E0 1,0E1 1,0E2 1,0E3 1,0E4 1,0E5 1,0E6
Titre
IPV Visit 1
IPV Visit 4
IPV-Al Visit 1
IPV-Al Visit 4

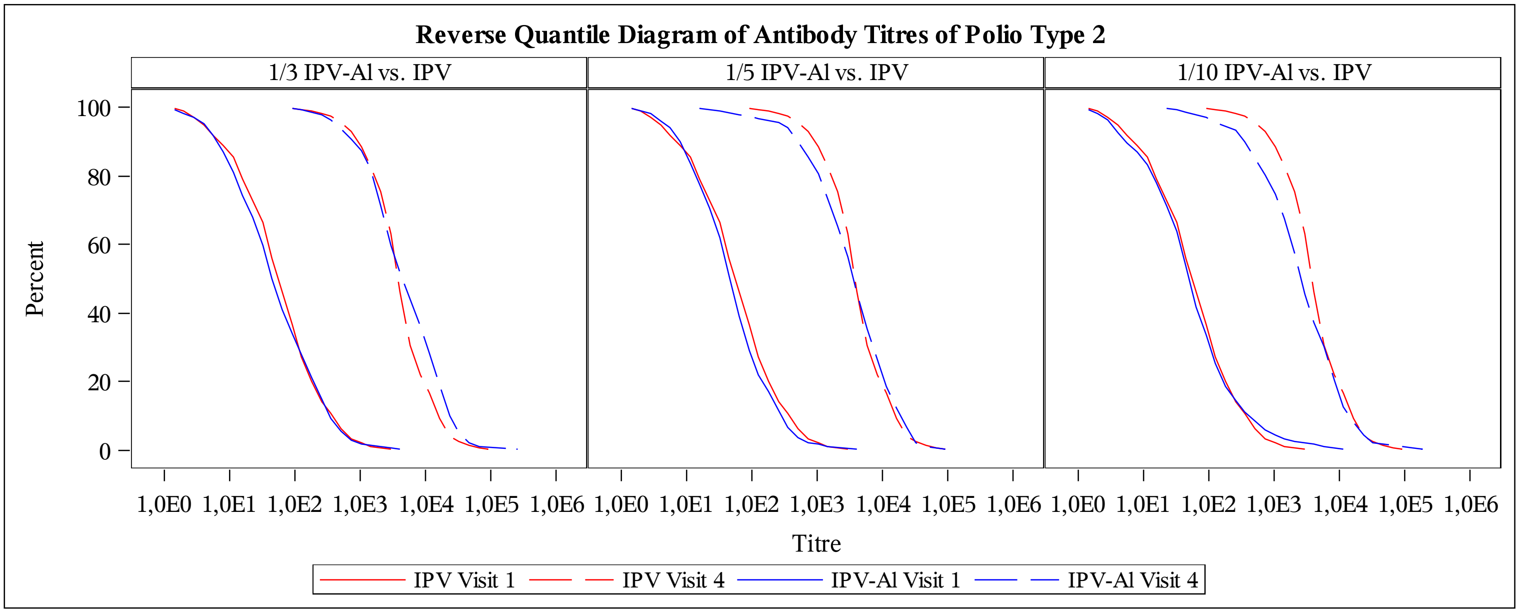
Reverse Quantile Diagram of Antibody Titres of Polio Type 2
1/3 IPV-Al vs. IPV
1/5 IPV-Al vs. IPV
1/10 IPV-Al vs. IPV
Percent
100
80
60
40
20
0
1,0E0 1,0E1 1,0E2 1,0E3 1,0E4 1,0E5 1,0E6
Titre
IPV Visit 1
IPV Visit 4
IPV-Al Visit 1
IPV-Al Visit 4

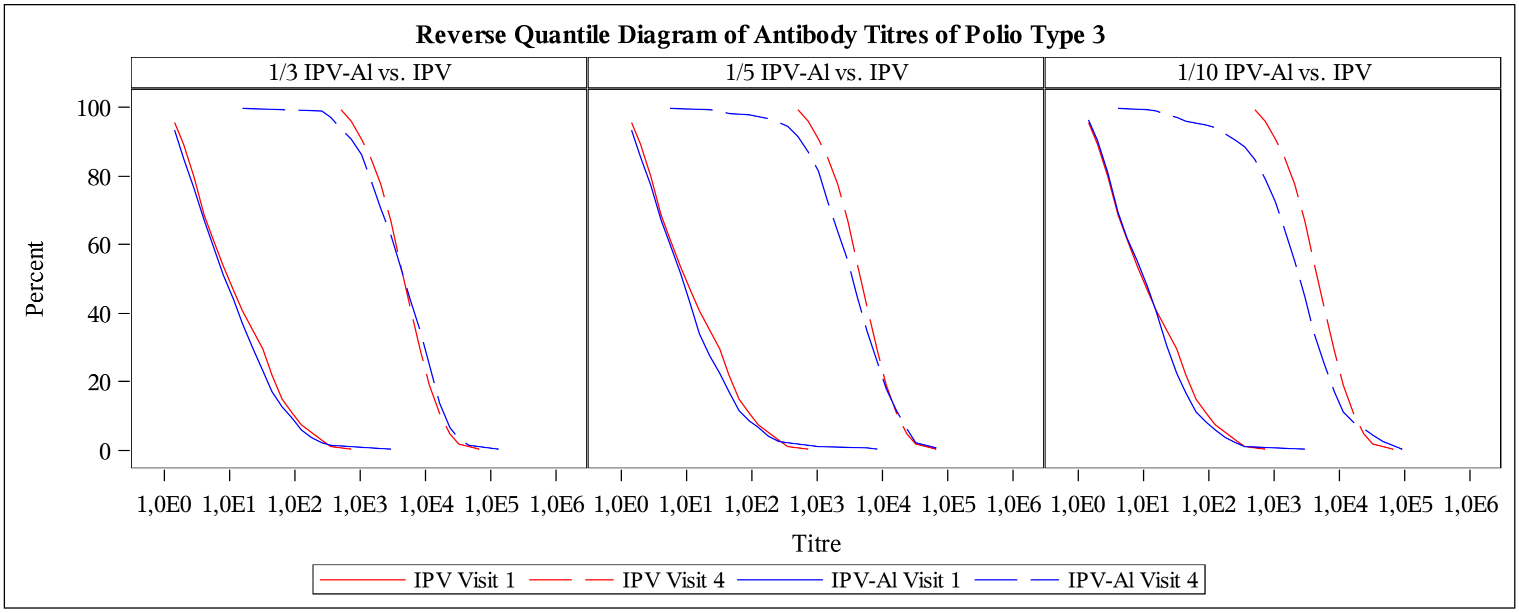

Reverse Quantile Diagram of Antibody Titres of Polio Type 3
1/3 IPV-Al vs. IPV
1/5 IPV-Al vs. IPV
1/10 IPV-Al vs. IPV
Percent
100
80
60
40
20
0
1,0E0 1,0E1 1,0E2 1,0E3 1,0E4 1,0E5 1,0E6
Titre
IPV Visit 1
IPV Visit 4
IPV-Al Visit 1
IPV-Al Visit 4
